# An evaluation of the AM 721 ion-selective electrode system for the estimation of sodium and potassium in plasma, urine and whole blood

**DOI:** 10.1155/S1463924683000450

**Published:** 1983

**Authors:** Peter West

**Affiliations:** Department of Clinical Biochemistry North Middlesex Hospital Edmonton London N18 1QX UK


Journal of Automatic Chemistry, Volume 5, Number 4 (October-December 1983), pages 182-187

### An evaluation of the AM 721 ion-selective electrode system for the estimation of sodium and potassium in plasma, urine and whole blood

**Peter West**

*Department of Clinical Biochemistry, North Middlesex Hospital, Edmonton, London N18 1QX*

### Introduction

In recent years, a number of different ion-selective electrodes for the measurement of sodium and potassium have been evaluated against existing methods of flame photometry with good agreement between the two methods in terms of precision and accuracy [1-6].

Recently, another ion-selective electrode instrument has been introduced—the AM 721 'Direct dip' system (Kontron Instruments Ltd, Campfield Road, St. Albans AL1 5JG, Hertfordshire, UK). This instrument is able to measure directly the ion concentration of both sodium and potassium in whole blood, plasma or urine. This paper reports an evaluation of the instrument and provides some comparative data with other systems.

### Description of the instrument

The AM 721 consists of four sections—a front panel with controls and indicators, a front-door assembly, an electrode chamber area, and a rear panel.

On the front panel are the calibration buttons and visual displays. The 'normal' and 'high' buttons are used for electrical check, calibration and measurement. Each button is pressed and released before taking a reading. The LED visual displays for the results are located above the calibration buttons.

The front-door assembly has a window to permit access to the electrode chamber area, part of this area being visible through the window of the front-door assembly.

In the electrode chamber area, the sample in the cup is vibrated and measured by electrodes with sensor tips. The three electrodes (a black reference, yellow sodium and red potassium electrode) are held in the electrode-holder portion of the electrode lift assembly. The electrode holder is connected by a shaft to the electrode lifter knob at the top of the instrument. This knob moves up, through a 30° rotation while up, and down, moving the electrodes to the right and left positions. When the knob is midway the electrodes are suspended out of solution.

On the rear panel are four labelled potentiometers; the $K^+$ and $Na^+$ potentiometers adjust the set points for the calibration. In the right-hand corner of the rear panel is a mode switch enabling the operator to select 'P' (whole blood or plasma) by switching to the lower position, or 'U' (urine) by switching to the upper position.

### Operation of the instrument

Samples are added to special disposable trays each of which is divided up into both a left- and right-hand cavity. The sample volume of 300 $\mu$l is dispensed into the trays using a special pipette: the 'Konnpipette'. Both the trays and Konnpipette are supplied with the AM 721.

A two-point calibration is carried out prior to initial use each day and after every 15 to 25 analyses. The operator chooses the mode of operation (P or U) as described above; if U is chosen, the U indicator will be visible on the front of the instrument.

300 $\mu$l of normal calibrant is dispensed into the right-hand cavity of the sample tray, and 300 $\mu$l of high calibrant into the left-hand tray. Separate calibrants are provided for plasma and urine.

After inserting the tray into the instrument, the electrodes are lowered into the normal calibrant, initiating a 15-25 s period of mixing between the sample and electrode assembly. Once mixing has stopped, time is allowed for stabilization (about 10 s) after which the 'normal' button is pressed and released.

Values are checked on a visual display to ensure that they are within the required limits. The electrodes are then lowered into the high calibrant and the process repeated with the high button pressed and released. If the values are within the required limits for this calibrant, the electrodes are returned to the normal calibrant and the process repeated—the values are checked without pressing the normal button. If calibration is successful, the operator proceeds to sample measurement. If not, the process is repeated using fresh calibrants. If this still proves unsuccessful, the operator is referred to the trouble-shooting section of the instruction manual.

### *Sample measurement*

Three hundred $\mu$l of normal calibrant is placed in the right cavity of a disposable tray and 300 $\mu$l of sample in the left cavity. The electrodes are placed in the normal calibrant and after mixing and stabilization, the normal button pressed. Once the values on the display have been checked, the electrodes are placed in the sample and the process repeated.

The results are read directly from the visual display. In the plasma mode, potassium results are given to two decimal places, in the urine mode, to one. No decimal places are given for sodium in either mode.

Once sample measurement is complete, the electrodes are placed in the normal calibrant, which automatically places the instrument in a standby mode. The electrodes lose calibration and may become damaged if left exposed to air.

### Special procedures for plasma and urine

In the event of there being an insufficient plasma volume (i.e. less than 300 μl) the operator may use the microsample procedure which needs only 50 μl. The normal and high calibrants, together with the samples, are diluted one in seven using the microsample dilutor solution in a test-tube; they are mixed well and then 300 μl of diluted normal calibrant is placed in the right-hand cavity of a disposable cup and the sensor tips of the electrodes placed in this for 15 min to equilibrate. A two-point calibration is carried out. The diluted sample is then measured and the results recorded.

A diluted urine mode procedure is also available. A one in seven dilution is made for the calibrants and samples using a different diluent to that used for plasma. Otherwise the procedure for urine is identical to that for plasma.

### Materials and methods

### Plasma

Blood was received in lithium heparin from 117 patients. The plasma was analysed on both the Technicon SMA 2 by flame photometry and on the AM 721.

Precision was assessed at three levels using commercial preparations—Wellcomtrols One, Two and Three (Wellcome Reagents, Wellcome Research Laboratories, Beckenham, Kent, UK).

Linearity was assessed using another commercial preparation—Hyland Q Pak 2 (Travenol Laboratories, Lessines, Belgium).

For sodium, this material was reconstituted with 4 ml of distilled water instead of the recommended 5 ml in order to give a sodium value of approximately 190 mmol/l. For potassium, the material was reconstituted with 3 ml distilled water to give a potassium value of approximately 10 mmol/l. Dilutions were made of the reconstituted preparations to cover the range 10–100% (potassium) and 40–100% (sodium).

Carry-over was assessed by the method of Young and Gochman [7]. Three successive identical samples with a high value (H1, H2 and H3) were followed by three successive identical samples with a low value (L1, L2 and L3). Percentage carry-over was calculated from the formula:

$$\frac{L1-L3}{H3-L3} \times 100.$$

Within-batch precision of the microsample procedure was assessed at three levels using patient samples, one with a high, one with a normal and one with a low level of sodium and potassium.

### Urine

Within- and between-batch-precision was assessed using Ortho Control Urine One (Ortho Diagnostic Systems, Raritan, New Jersey, USA).

Accuracy was assessed by comparing the results obtained on the AM 721 with those by a routine flame photometer method on a Technicon AA 2 system for 60 patient specimens. All urines were aliquots of 24 h collections in bottles containing thymol as preservative. An independent assessment of accuracy was made by comparing the values obtained for the Ortho Control Urine One with the manufacturer's target values.

### Whole blood

Blood samples were received in lithium heparin tubes. An aliquot was taken for analysis on the AM 721 and the sample then centrifuged in a closed tube at 2500 r.p.m. for 10 min in a non-thermostatted centrifuge. Plasma was normally measured on the AM 721 within 1 h of separation.

A recent study has shown the effect of different conditions of centrifugation on plasma sodium and potassium [9]. Whereas centrifugation speed and temperature (20°C compared with a non-thermostatted centrifuge) has no significant effect on the sodium and potassium content of plasma, the influence of closure of the centrifuge tube has. Centrifugation in open tubes may, due to the evaporation of plasma water, increase the sodium by as much as 1·5 mmol/l in 10 min.

Within-batch precision of the whole blood was made by repeat analysis of a number of patient samples.

A correlation between whole blood and plasma values was made in 80 patient samples.

### Results and discussion

### Plasma

#### Precision

Both the within- and between-batch precision data for the instrument are shown in table 1. Between-batch precision gave coefficients of variation below 1% for sodium and below 2% for potassium at all levels. Over the same period of evaluation as the AM 721, the Technicon control material run on the SMA 2 gave comparable between-batch coefficients of variation of 0·65% for sodium at a mean level of 148·1 mmol/l and 1·58% for potassium at a level of 5·6 mmol/l.

*Table 1. Precision data for plasma sodium and potassium on the AM 721 ion-selective electrode instrument (in mmol/l).*

| | Sodium | | Potassium | |
|---|---|---|---|---|
| Control | Within-batch | Between-batch | Within-batch | Between-batch |
| Wellcomtrol One | | | | |
| Number | 20 | 46 | 20 | 46 |
| Mean | 140·30 | 140·51 | 4·72 | 4·63 |
| SD | 0·82 | 1·15 | 0·04 | 0·08 |
| CV(%) | 0·58 | 0·82 | 0·85 | 1·73 |
| Wellcomtrol Two | | | | |
| Number | 20 | 44 | 20 | 44 |
| Mean | 145·40 | 146·84 | 5·54 | 5·52 |
| SD | 0·97 | 1·23 | 0·07 | 0·07 |
| CV (%) | 0·67 | 0·84 | 1·26 | 1·27 |
| Wellcomtrol Three | | | | |
| Number | 20 | 45 | 20 | 45 |
| Mean | 131·30 | 130·47 | 3·99 | 3·96 |
| SD | 0·48 | 1·24 | 0·03 | 0·06 |
| CV (%) | 0·37 | 0·95 | 0·75 | 1·52 |

#### Accuracy

Figures 1 and 2 show the results for 117 patient plasmas analysed on both instruments. Potassium results on the AM 721 were corrected to one decimal place to directly compare them with those obtained by routine analysis.

The correlation coefficients for sodium and potassium were 0·95 and 0·97 respectively. Sodium gave results which were on average 1·85 mmol/l lower, potassium results on average 0·16 mmol/l higher than those obtained by routine flame

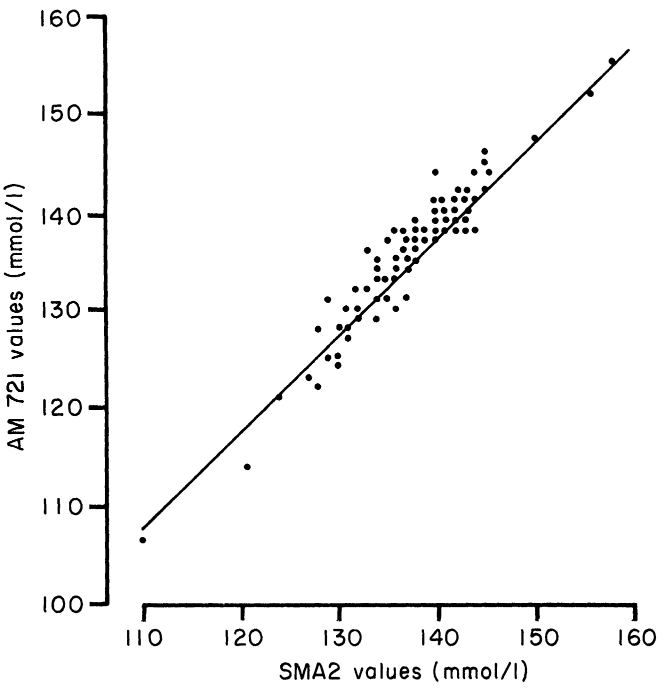

*Figure 1. Comparison of results obtained in 117 patient samples by the AM 721 and flame photometry on the SMA 2 for plasma sodium, $r=0{\cdot}95$, $y=1{\cdot}03X-5{\cdot}9$ (where y = AM 721 value, x = SMA 2 value, units are mmol/l).*

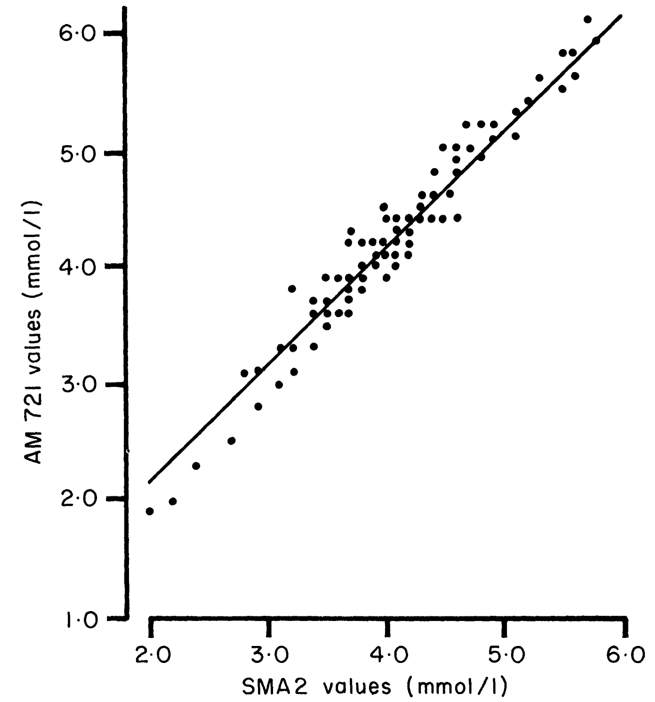

*Figure 2. Comparison of results obtained in 117 patient samples by the AM 721 and flame photometry on the SMA 2 for plasma potassium $r=0{\cdot}97$, $y=1{\cdot}03X+0{\cdot}1$ (where y = AM 721 values, x = SMA 2 value, units are mmol/l).*

photometry. The lower sodium results are in contrast to those reported on other ion-selective electrodes, where, in general, sodium was found to produce results which were higher than by flame photometry [2–6]. The higher potassium results on the AM 721 are in agreement with results obtained on other instruments [2–6].

It has recently been suggested that laboratories should determine their own correlation between their flame photometer and ion-selective electrode results and correct the ion-selective electrode values to those obtained by flame photometry [8]. An alternative approach might be to obtain a different reference range for the ion-selective electrodes.

A further assessment of accuracy was made by comparing the overall mean values obtained using Wellcomtrols One and Three with target values quoted by the manufacturer for ion-selective electrode methodology. (No value was quoted for Wellcomtrol Two.) The comparison is shown in table 2. Values obtained on the AM 721 agreed well with the target values.

*Table 2. Comparison of the results obtained on the AM 721 with the target values quoted by the manufacturer for ion-selective methodology (in mmol/l).*

| | Sodium | | Potassium | |
|---|---|---|---|---|
| Control | AM 721 | Target | AM 721 | Target |
| Wellcomtrol One | 140·50 | 139·90 | 4·63 | 4·81 |
| Wellcomtrol Three | 130·47 | 131·0 | 3·96 | 3·95 |

*Linearity*

Linearity data for sodium and potassium are shown in figures 3 and 4 respectively. Linearity for sodium was found to fall off below 90 mmol/l; potassium was found to be linear between 1 and 10 mmol/l. The range was greater than that quoted for the Orion SS 30 analyser for both sodium and potassium [3] and the upper limit of the range for potassium was greater than that found on the Nova 1 [6].

*Carry-over*

The mean carry-over, which was assessed on three occasions, was found to be 0·6% for sodium and 0·4% for potassium.

*Precision of the microsample procedure*

Within-batch precision data at three levels of sodium and potassium are given in table 3. These figures were obtained using enough diluted normal calibrant for both the calibration and precision study.

### *Urine*

Initial studies using the undiluted urine mode showed some large discrepancies between results obtained on the AM 721 and by routine flame photometry. This was particularly evident for potassium; values for the control urine were close to the target values. The control was reconstituted in distilled water and the urine samples were collected in thymol. The instruction manual states that certain preservatives may affect the performance of the electrodes, but this statement is not elaborated. The problem was solved by using the diluted urine procedure.

*Precision*

The within-batch and between-batch precision data for the diluted urine mode are shown in table 4. Normal calibrant was diluted in bulk for the purpose of the within-batch study and for each batch of patient or between-batch quality-control samples.

Between-batch precision data obtained over the same period of evaluation as the AM 721 by the routine flame photometer method using a pooled control urine gave a CV of 2·33% for sodium (a mean of 125·9 mmol/l) and 2·52% for potassium (a mean of 61·2 mmol/l). The slightly higher figures for the AM 721 may in part be due to the manual dilution step prior to analysis.

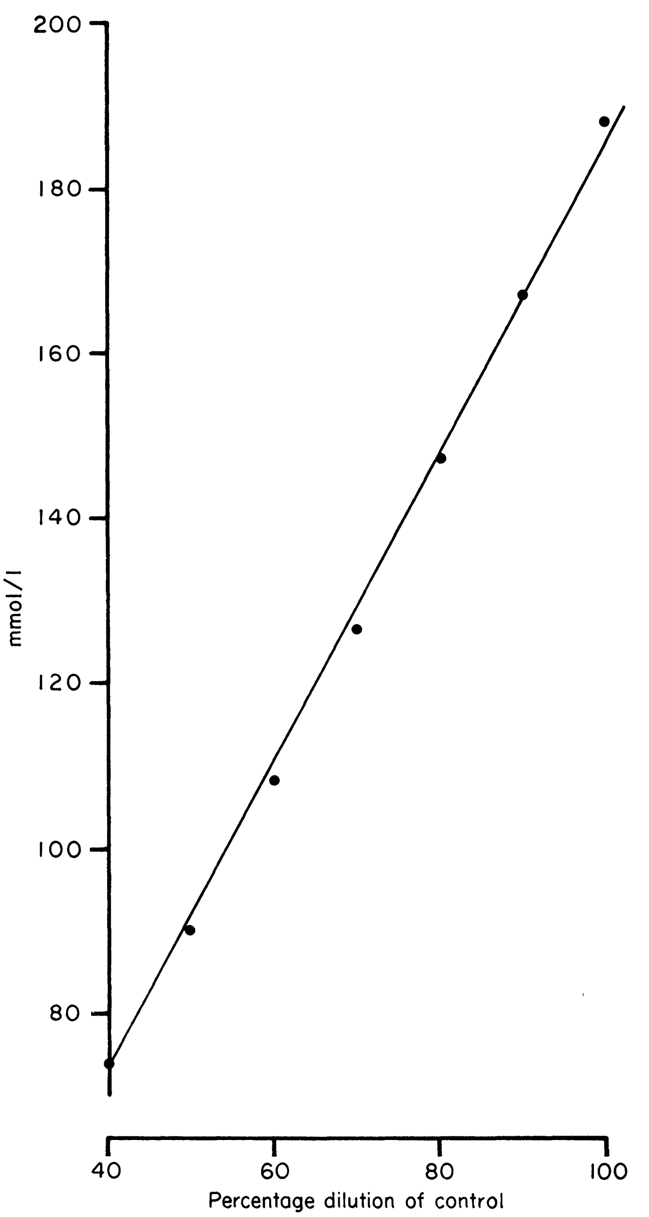

*Figure 3. Linearity data for plasma sodium. The y axis gives the value in mmol/l, the x axis the dilution of the control material, ranging from 40 to 100%.*

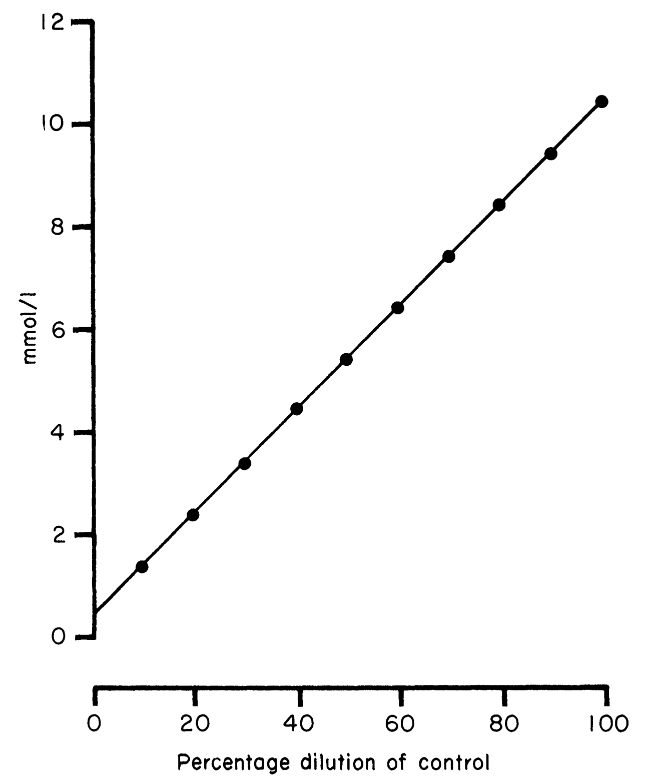

*Figure 4. Linearity data for plasma potassium. The y axis gives the value in mmol/l, the x axis, the dilution of the control material, ranging from 10 to 100%.*

*Table 3. Within-batch precision for plasma sodium and potassium using the special microsample dilution procedure (in mmol/l).*

| | Sodium | Potassium |
|---|---|---|
| *Low level* | | |
| Number | 20 | 20 |
| Mean | 120·18 | 2·56 |
| SD | 1·94 | 0·06 |
| CV (%) | 1·61 | 2·34 |
| *Normal level* | | |
| Number | 20 | 20 |
| Mean | 136·33 | 3·65 |
| SD | 0·71 | 0·05 |
| CV (%) | 0·52 | 1·37 |
| *High level* | | |
| Number | 20 | 20 |
| Mean | 150·17 | 5·32 |
| SD | 1·72 | 0·08 |
| CV (%) | 1·15 | 1·50 |

*Table 4. Precision data for urine sodium and potassium. These data were obtained using the diluted urine mode procedure (in mmol/l).*

| | Sodium Within-batch | Potassium Within-batch | Sodium Between-batch | Potassium Between-batch |
|---|---|---|---|---|
| Number | 20 | 20 | 20 | 20 |
| Mean | 90·84 | 36·42 | 89·28 | 35·35 |
| SD | 0·76 | 0·58 | 2·27 | 1·34 |
| CV (%) | 0·84 | 1·60 | 2·54 | 3·79 |

*Accuracy*

Figures 5 and 6 show the results for 60 urine samples analysed on both instruments. Potassium results were taken to the nearest whole number to directly compare them with those obtained by routine analysis. The correlation coefficients for sodium and potassium were 0·99 and 0·98 respectively. Sodium produced results on the AM 721 which were on average 0·5 mmol/l lower than flame photometry; potassium results were on average 2·6 mmol/l lower than those obtained by flame photometry. A similar finding of a greater discrepancy for potassium was found on the Nova 1 [6].

An independent assessment of accuracy was made by comparing results obtained for the control urine with the target value quoted by the manufacturer for the control material. Unlike the Wellcomtrols, no target value was quoted for ion-selective electrode methodology, so a comparison was made of values quoted for the SMA 6/60 flame photometer method. The overall mean value for sodium (89·28) was 1·5% higher, and the potassium value (35·35) was 1% lower than the target value quoted.

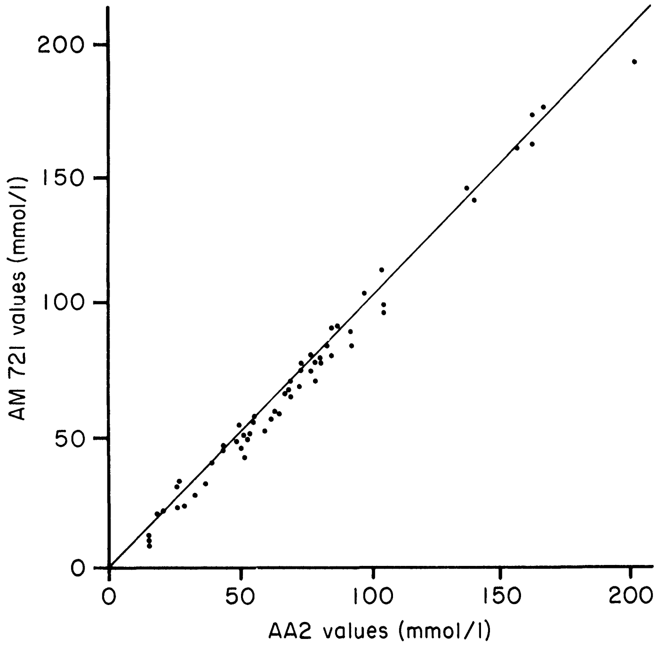

*Figure 5. Comparison of results obtained in 60 patient samples by the AM 721 and flame photometry on the AA 2 for urine sodium.* $r=0{\cdot}99$, $y=1{\cdot}02x-2{\cdot}2$ *(where* $y$ *= AM 721 value,* $x$ *= AA 2 value, units are mmol/l).*

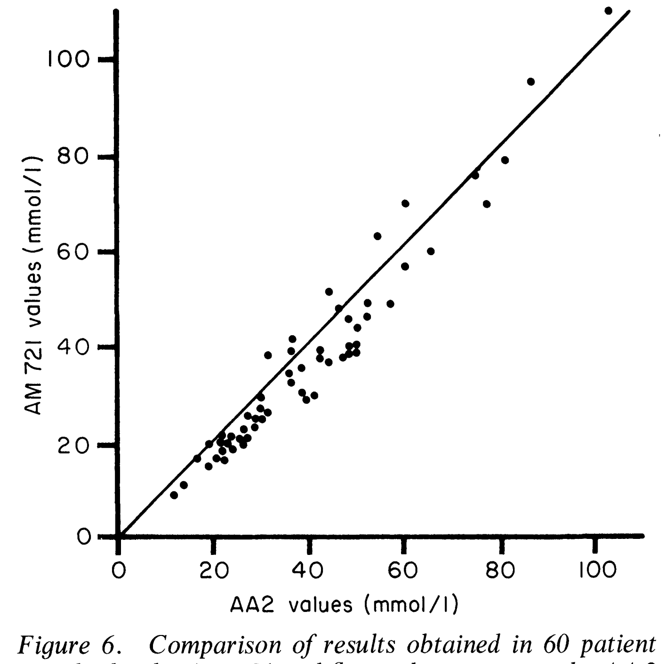

*Figure 6. Comparison of results obtained in 60 patient samples by the AM 721 and flame photometry on the AA 2 for urine potassium.* $r=0{\cdot}98$, $y=1{\cdot}06X-5{\cdot}0$ *(where* $y$ *= AM 721 value,* $x$ *= AA 2 value, units are mmol/l).*

### *Whole blood*

*Precision*

Within-batch precision for whole blood for seven patient samples is shown in table 5. Each figure represents the mean of 10 analyses.

*Comparison with plasma*

The results for 80 patient samples comparing the whole blood and corresponding plasmas are given in table 6. The potassium results were taken to one decimal place. The overall correlation coefficients for sodium and potassium were 0·96 and 0·98 respectively.

*Table 5. Within-batch precision data for the whole blood mode for seven patient samples. Each sample was analysed 10 times (in mmol/l).*

| Sodium | | | Potassium | | |
|---|---|---|---|---|---|
| Mean | SD | CV (%) | Mean | SD | CV (%) |
| 141·40 | 0·70 | 0·50 | 3·74 | 0·05 | 1·34 |
| 138·90 | 0·57 | 0·41 | 5·10 | 0·06 | 1·18 |
| 112·20 | 1·47 | 1·31 | 2·38 | 0·04 | 1·68 |
| 135·56 | 0·52 | 0·38 | 3·31 | 0·03 | 0·91 |
| 141·00 | 0 | 0 | 4·15 | 0·05 | 1·20 |
| 142·30 | 0·82 | 0·5 | 5·61 | 0·06 | 1·07 |
| 124·20 | 0·44 | 0·35 | 4·37 | 0·05 | 1·14 |

*Table 6. Data obtained in 80 samples comparing whole blood and plasma measurements (y = plasma value and x = whole blood value). The mean whole blood value (mmol/l) refers to the bias found in blood when compared with measurement in plasma.*

| Analyte | Correlation coefficient | Equation of the line of best fit | Mean whole blood value (mmol/l) |
|---|---|---|---|
| Sodium | 0·96 | $y=0{\cdot}95\,x+6{\cdot}91$ | −0·15 |
| Potassium | 0·98 | $y=0{\cdot}99\,x+0{\cdot}02$ | +0·01 |

The range of values covered in whole blood for sodium was 112 to 145 mmol/l, and 2·6 to 5·6 mmol/l for potassium. Sodium results in whole blood were, on average, 0·15 mmol/l lower than those found in plasma (mean whole blood sodium 135·11 mmol/l, mean plasma sodium 135·26 mmol/l). Potassium results in whole blood were, on average, 0·01 mmol/l higher than those found in plasma (mean whole blood potassium 3·94 mmol/l, mean plasma potassium 3·93 mmol/l). From the results, comparing whole blood and plasma, it seems probable that no separate reference range needs to be established for whole blood sodium and potassium.

The design of the reference electrode has been shown to be an important factor in determining whether differences between whole blood and plasma are significant or not [9].

### General comments

### *Instruction manual*

The manual provided with the AM 721 was simple to follow. It is divided into several sections including ones on how to adjust the potentiometer set points and trouble-shooting (problem-solving). The trouble-shooting section lists four groups of problems which might be encountered. These are listed in the approximate order in which they would occur in going from installation to calibration to sample measurement. The problems are analysed in two columns, based on whether the problem is shown by both the sodium and potassium electrode or by only one of the electrodes. Each column lists the common causes and solutions.

The comment at the beginning of the manual on the possible effect of urine preservatives on the electrode performance was found to be relevant in the present study, and could have been usefully restated in the section on urine measurement. Each laboratory should evaluate the effect of their urine preservative (if one is used) on the performance of the electrodes before proceeding with this mode.

### *Costs*

The approximate cost of the instrument at the time of the evaluation was £2430 plus value-added tax. The reference and sodium electrodes cost approximately £110 each, the potassium electrode £70; a boxed set of all three electrodes is priced at £230. The disposable cups, supplied in batches of 300, are around £0·05 each. It is possible to reuse them by soaking overnight in decon solution, followed by rinsing in water and drying in the oven prior to reuse, and this can bring down cost per test by a considerable amount. The normal and high calibrants for plasma and whole blood are supplied in bottles of 58 ml each at around £12·00 per bottle. Urine calibrants and one bottle of urine dilutor solution come as a set, each set costing about £12·00 (each of the three bottles contains 118 ml of solution). The microsample dilutor solution is supplied in a box, each box containing two 118 ml bottles.

### General conclusions

The instrument was found to be simple to operate with results obtainable within 70–75 s of starting to sample the calibrant and specimen.

The visual display on the instrument was clear and generally easy to read—it was difficult in bright sunlight.

The lifetime of the sodium electrode is claimed to be four years and the potassium electrode is one year. The lifetime depends to some extent on adequate maintenance of the electrode system. Daily cleaning in a solution of 0·1 M ammonium bifluoride is recommended—this solution is provided with the instrument.

The instrument shares with similar systems the advantage of safety over flame photometry, as no gas or compressed air supply is required. Additional advantages which the author feels the instrument has over other systems include the size of the instrument and the volume of sample required for assay. The AM 721 weighs only 5 kg and its dimensions are 28 cm in height and 25 cm in width. Comparable figures for one other instrument, the Nova 1, are 43 kg weight, 48·4 cm height and 39·4 cm width. Size can often be an important factor in a clinical chemistry laboratory where space may not be readily available. An insufficient volume of plasma (i.e. less than 300 $\mu$l) may be encountered, especially in paediatric samples. Here, the operator may use the special microsample procedure requiring only 50 $\mu$l; the Orion SS 30 uses 500 $\mu$l of plasma and the Nova 1 needs 180 $\mu$l. The instrument would be most useful as an out-of-hours sodium and potassium analyser for clinical chemistry laboratories. It could also find a home in intensive-care units, surgical and renal units, or out-patient departments where urgent results may be needed.

A minimum of skill is needed to operate the instrument. The study has shown that there is good agreement between whole blood and plasma. Therefore in units where a centrifuge is not always available, whole blood analysis can be performed (the only requirement being a source of electricity).

### Acknowledgements

I should like to thank Mike Coates, Clinical Chemistry Division, Kontron Ltd for his help during the evaluation; Robert Wilson, Principal Biochemist, North Middlesex Hospital for helpful comments on the paper; Dr Gerald Levin, Senior Lecturer in Chemical Pathology, St. George's Hospital Medical School for arranging for the diagrams to be drawn and Miss Deborah Young for typing the script.

### References

1. Lustgarten, J. A., Wenk, R. E., Byrd, C. and Hall, B., *Clinical Chemistry*, **20** (1974), 217.
2. Uddin, De, Hickey, T. M. and Fisher, S. A., *Clinical Chemistry*, **23** (1977), 1129. Abstract No. 41.
3. Kozak, C. and Kolansinski, K., *Clinical Chemistry*, **23** (1977), 1125. Abstract No. 42.
4. Patel, S. and O'Gorman, P., *Clinical Chemistry*, **24** (1978), 1856.
5. Ladensen, J. W., *Clinical Chemistry*, **25** (1979), 757.
6. Annan, W., Kirwan, N. A., Robertson, W. A., Teasdale, P. R. and Ager, B. P., *Journal of Automatic Chemistry*, **2** (1980), 212.
7. Young, D. S. and Gochman, N., *Standard Methods in Clinical Chemistry*, **7** (1972), 293.
8. Maas, A. H. J., Siggaard-Andersen, O., Weissberg, H. and Zijlstra, W., *International Federation of Clinical Chemistry Newsletter*, No. 31 (1982).
9. Bijster, P., Vader, H. L. and Vink, C. L. J., *Annals of Clinical Chemistry*, **20** (1983), 116.

### IUPAC 1985

*The 30th International Congress of Pure and Applied Chemistry*

The 30th IUPAC Congress will be held from Monday 9 to Friday 13 September 1985 in Manchester, UK. The opening ceremony and the inaugural plenary lecture will take place on Monday morning; the meeting will end at lunchtime on Friday. A full programme of evening and daytime social events is being planned.

The Royal Society of Chemistry is responsible for the detailed organization of the Congress.
Prominent chemists from all over the world are being invited to present lectures. The symposia within the individual sections will take place both concurrently and sequentially. It is planned to include free-offer contributions, particularly as posters. Details regarding submission of papers will be notified in a circular, which will be available in March 1984.

*Requests to be added to the mailing list for circulars to the Royal Society of Chemistry, Burlington House, London W1V 0BN.*

